# The Heterogeneity and Spatial Patterning of Structure and Physiology across the Leaf Surface in Giant Leaves of *Alocasia macrorrhiza*

**DOI:** 10.1371/journal.pone.0066016

**Published:** 2013-06-11

**Authors:** Shuai Li, Yong-Jiang Zhang, Lawren Sack, Christine Scoffoni, Atsushi Ishida, Ya-Jun Chen, Kun-Fang Cao

**Affiliations:** 1 School of Life Sciences, University of Science and Technology of China, Hefei, Anhui Province, China; 2 Key Laboratory of Tropical Forest Ecology, Xishuangbanna Tropical Botanical Garden, Chinese Academy of Sciences, Menglun, Mengla, Yunnan Province, China; 3 Department of Organismic and Evolutionary Biology, Harvard University, Cambridge, Massachusetts, United States of America; 4 Department of Ecology and Evolutionary Biology, University of California Los Angeles, Los Angeles, California, United States of America; 5 Center for Ecological Research, Kyoto University, Otsu, Shiga, Japan; 6 Graduate University of Chinese Academy of Sciences, Beijing, China; 7 State Key Laboratory for Conservation and Utilization of Subtropical Agro-Bioresources, The Key Laboratory of Ministry of Education for Microbial and Plant Genetic Engineering, and College of Forestry, Guangxi University, Nanning, Guangxi Province, China; UMass, United States of America

## Abstract

Leaf physiology determines the carbon acquisition of the whole plant, but there can be considerable variation in physiology and carbon acquisition within individual leaves. *Alocasia macrorrhiza* (L.) Schott is an herbaceous species that can develop very large leaves of up to 1 m in length. However, little is known about the hydraulic and photosynthetic design of such giant leaves. Based on previous studies of smaller leaves, and on the greater surface area for trait variation in large leaves, we hypothesized that *A. macrorrhiza* leaves would exhibit significant heterogeneity in structure and function. We found evidence of reduced hydraulic supply and demand in the outer leaf regions; leaf mass per area, chlorophyll concentration, and guard cell length decreased, as did stomatal conductance, net photosynthetic rate and quantum efficiency of photosystem II. This heterogeneity in physiology was opposite to that expected from a thinner boundary layer at the leaf edge, which would have led to greater rates of gas exchange. Leaf temperature was 8.8°C higher in the outer than in the central region in the afternoon, consistent with reduced stomatal conductance and transpiration caused by a hydraulic limitation to the outer lamina. The reduced stomatal conductance in the outer regions would explain the observed homogeneous distribution of leaf water potential across the leaf surface. These findings indicate substantial heterogeneity in gas exchange across the leaf surface in large leaves, greater than that reported for smaller-leafed species, though the observed structural differences across the lamina were within the range reported for smaller-leafed species. Future work will determine whether the challenge of transporting water to the outer regions can limit leaf size for plants experiencing drought, and whether the heterogeneity of function across the leaf surface represents a particular disadvantage for large simple leaves that might explain their global rarity, even in resource-rich environments.

## Introduction

Leaves first appeared in terrestrial plants about 400 million years ago and arose independently multiple times in a number of plant lineages, eventually evolving an enormous diversity in shape, size, internal structure and longevity across environments [1], [2], [3], [4], [5]. Leaves sustain the whole plant through photosynthesis, the biochemical and physiological processes of light capture, carbon fixation, and synthesis of carbohydrates [6], [7]. However, leaves lose water through transpiration during photosynthesis and must invest in replacing the lost water or suffer desiccation [8]. Indeed, leaves represent a bottleneck in the plant hydraulic system [9], [10], and their venation network must be adequate to supply enough water for a given stomatal pore area and given gas exchange rate [10], [11], [12], [13]. The design of very large leaves may be especially challenging given that water must be transported from the petiole to the edges of the leaf via the xylem, which has substantial hydraulic resistance.

Consistent with the challenges of delivering water across the leaf surface, within a given leaf, structure and function can be heterogeneous [14], [15], [16]. Previous studies have shown variable patterns, e.g., that abaxial stomatal density increases continuously from the leaf base to tip in wheat [14], and that stomatal density decreases from the leaf center to edges in some eudicotyledons, including other monocots [16], [17]. These results suggest non-uniform water loss rates over a leaf surface [18]. Indeed, there is a marked heterogeneity of stomatal conductance and gas exchange rate across the leaf surface in tobacco leaves [16], corresponding with a greater vein density (i.e., vein length per leaf area) near the leaf tip than at the leaf base [16].

However, little is known of the hydraulic and photosynthetic design of giant leaves. Based on the previous studies of smaller leaves described above, and on the greater surface area for trait variation in large leaves, we hypothesized that the heterogeneity of structure and function might be very strong in a species such as *Alocasia macrorrhiza* (L.) Schott. (Araceae), a perennial herbaceous species which bears giant leaves of up to 1–2 m in length and width. This species provides a model system for studying the heterogeneity of leaf structure and function within a large leaf.

In giant leaves, hydraulic limitations in the outer regions of the leaf may considerably increase the risks of over-heating and chronic photo-inhibition. Indeed, leaf temperature is inversely correlated with stomatal conductance and transpiration rate [19], and stomatal closure results in an elevated leaf temperature [20], [21], which in turn can inhibit photosynthesis [22]. Temperatures slightly higher than those optimal for growth can inhibit or damage photosystem II (PSII) [23], [24], and the combination of high light and high temperature leads to a more severe and sustained photoinhibition than either stress alone [25], [26], [27], [28]. Indeed, the combination of high light and elevated leaf temperature has been found to decrease Rubisco activity in *A. macrorrhiza* and other species [27], [29]. Therefore, lower stomatal conductance at the leaf edges would lead directly to lower rates of gas exchange, which may induce further closure by increasing leaf temperature causing an even greater reduction in photosynthesis.

We tested the hypothesis that the heterogeneity in leaf structure and function would contribute to higher temperatures and a lower gas exchange rate in the outer lamina regions, given increased hydraulic path length and resistance in these distal parts. Such an effect could be particularly strong in large leaves, and may correspond with greater stomatal closure and/or the development of fewer and/or smaller stomata, and thus induced a lower photosynthetic rate in the leaf outer regions. To test this hypothesis, we examined stomatal conductance, photosynthetic rate and PSII function, as well as leaf water potential, and leaf temperature at midday, as well as leaf structural and anatomical traits, along length and width transects from the center to the outer regions within the large leaves of *A. macrorrhiza*.

## Materials and Methods

### Study Site and Plant Material

The study was conducted at the Xishuangbanna Tropical Botanical Garden (21° 41′ N, 101° 25′ E, and elevation 570 m), Chinese Academy of Sciences, in southern Yunnan, China. This region is at the northern margin of the Asian tropics, with a mean annual temperature of 21.7°C and a mean annual precipitation of 1560 mm. More than 85% of annual precipitation occurs from the May to October. Measurements were made in the wet period from July through August 2010. However, the high temperatures experienced on sunny days in the wet season can cause as much leaf water stress as the dry season in this system, especially because in the first four months of the dry season there is a heavy fog from midnight to noon almost every day, maintaining high soil moisture over much of the dry season.

### Leaf Anatomy and Morphology

**Figure 1:**
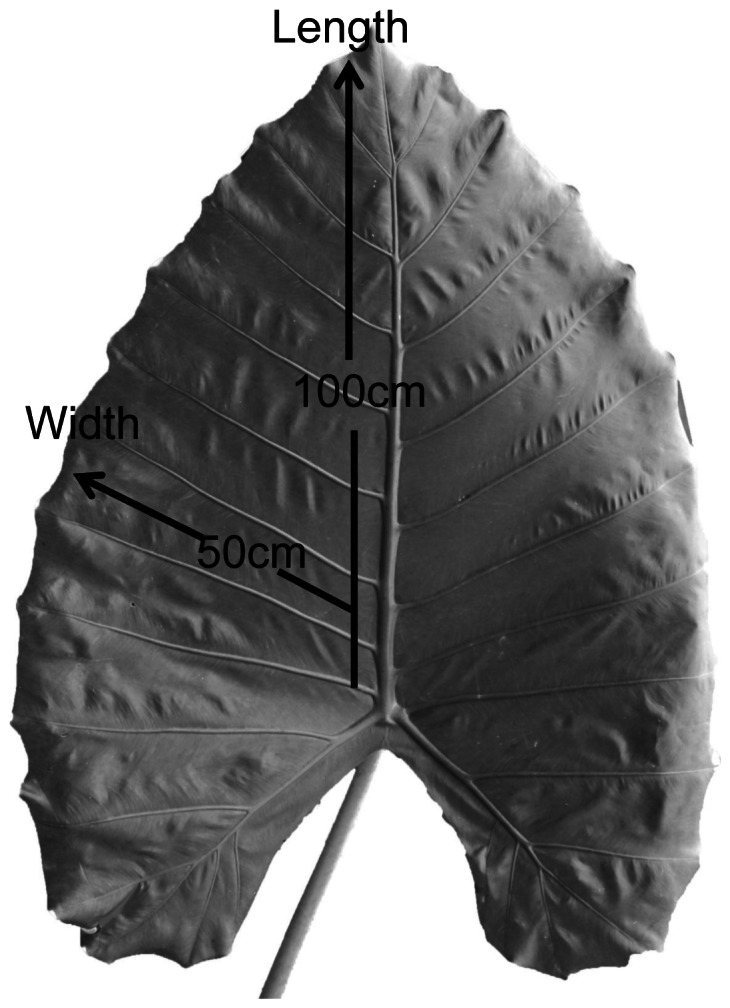
A typical leaf of *Alocasia macrorrhiza (L.) Schott*, showing the two transect directions for anatomical and physiological measurements.

To characterize the heterogeneity of structure and function within *A. macrorrhiza* leaves, we chose six mature and healthy individuals similar in leaf size, leaf age and environmental conditions, and selected one leaf from each individual that was fully exposed to sunlight. The *A. macrorrhiza* leaves were excised with a razor blade and brought to the laboratory. Leaf discs of 4 cm^2^ were sampled along transects in two directions: from the basal to apical lamina regions and from the central to outer lamina regions (Fig. 1). Leaf dry mass per area (LMA) was determined from the leaf disc area, and dry mass after oven-drying for 48 h at 70°C.

Using other leaf discs from the same leaves, chlorophyll concentration was determined using a portable chlorophyll meter (SPAD-502, Minolta CO., Japan), which provides values in SPAD units linearly correlated with total (a+b) chlorophyll concentration in g cm^−2^ [30]. We additionally determined leaf thickness and the ratio of palisade- to spongy- tissue thickness (P/S ratio). Leaf anatomical characteristics were measured from six hand-cut transverse sections of each leaf disc under a microscope (Leica DM2500, Leica Inc, Buffalo, NY, USA), imaged using a digital camera (Leica DFC295, Leica Inc, Buffalo, NY, USA), and measured using an image analysis software (Image J, National Institutes of Health, Bethesda, MD, USA). Abaxial epidermal impressions were made with clear nail polish and the guard cell length and stomatal density were determined. To measure vein density (i.e., vein length per leaf area), the epidermis was removed with a sharp razor blade and the remaining leaf samples were cleared in bleach (LIBY Enterprise Group Co. Ltd., Guangdong, China). The leaf samples were stained with toluidine blue to resolve and quantify vein length per area using Image J.

### Leaf Gas Exchange

Measurements of positional variation in leaf gas exchange across leaves were made at 1230 h and 1400 h on sunny days during the wet season for six sun-exposed mature and healthy leaves from six different individuals. The net CO_2_ assimilation per leaf area (*A*) and stomatal conductance to water vapor (*g*_s_) were measured with a portable photosynthesis system (Li-6400, Li-Cor Inc., Lincoln, NE, USA). Measurements were made with chamber CO_2_ concentration set to 400 µmol mol^−1^ and under ambient irradiance (>1000 µmol m^−2^ s^−1^). We began the gas exchange measurements at the outer or apical regions, and then made 2–3 additional measurements progressively towards the center or basal regions of the leaves, along the directions shown in Fig. 1. To allow measurements to be made using the instrument chamber, we progressively cut and removed lamina from the outer/apical region, while avoiding first- and second- order veins. The time duration between these treatments and measurements was kept as short as possible, to allow comparable measurements across the leaf and avoid any secondary effects of the leaf cutting. Each gas exchange measurement was made within 1 to 2 minutes and all the measurements along a direction were completed within 8 minutes.

### Leaf Water Potential

Measurements of positional variation across leaves in midday leaf water potential (Ψ_md_) were made with a dewpoint hygrometer (WP4, Decagon Devices, Inc., Pullman, WA, USA) on sunny days in the wet season. Seven leaf discs of 16 cm^2^ were sampled along the two different directions (Fig. 1) from each of six leaves of six individuals at 1230 h to 1400 h. The leaf discs were excised from the plants with a razor blade, immediately sealed in plastic bags and kept in a plastic box with moist paper towels and, once in the laboratory, were rapidly cut into pieces to accelerate the equilibration between the sample and the air in the sample cups. The WP4 hydrometer was set in continuous-mode and the equilibrium water potentials of the leaf samples were achieved in 20 to 30 minutes.

### Chlorophyll Fluorescence

Chlorophyll fluorescence measurements were conducted between 1230 h and 1400 h on sunny days in the rainy season, with a portable fluorescence monitoring system (FMS2, Hansatech Instrument Ltd., Norfolk, UK). The quantum yield of PSII (Φ_PSII_) was determined at 3–4 different positions on sun exposed leaves along the two directions. Incident PPFD measured with a quantum sensor attached to the leaf clip holder ranged from 699.5 to 1184.5 µmol m^−2^. The quantum yield of PSII was calculated as (*F*_m_’–*F*_s_)/*F*_m_ *’* (*F*_s_ = stationary level of fluorescence emission and *F*_m_’ = maximum fluorescence during illumination).

### Leaf Temperature

To determine the variation of leaf temperature across the leaves, infrared images were taken with a FLIR Infrared Camera (FLIR ThermaCAM-P25, FLIR Systems Inc, USA) from 1200 h to 1400 h on sunny days for six sun-exposed leaves. The camera was positioned at a small deviation from perpendicular to the leaf being inspected to avoid reflection. Using the instrument software (ThermaCAMQuickreport 1.1, FLIR systems Inc, USA) we analyzed images, adding a gradient of colors to represent a range of temperatures. Leaf temperature was also measured every minute with thermocouples (0.075 mm in diameter) connected to a data logger (CR10X, Campbell Scientific, Leicester, UK) at three positions along the lamina in the direction of the midrib and at three positions along the lamina in the direction of the secondary vein for six sun-exposed leaves. Thermocouples were attached to the abaxial surface of lamina with 4 cm^2^ pieces of porous athletic tape (medical porosity gummed tape, Yunnan Xiangshan Medical Material Co. Ltd., Yunnan, China) selected to minimize the inhibition of leaf gas exchange.

## Results

### Within-leaf Variation in Anatomy and Morphology

**Figure 2:**
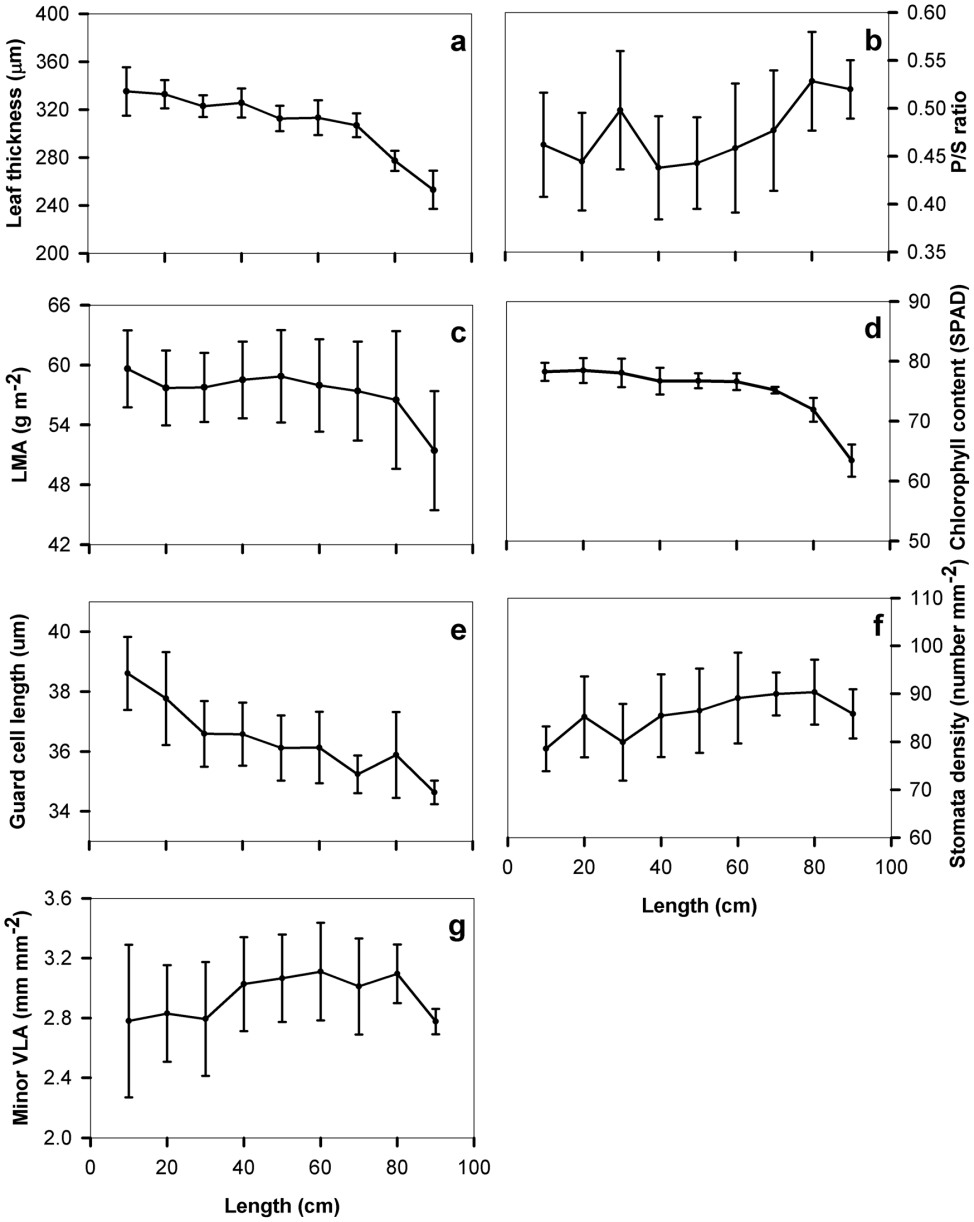
Leaf anatomical and structural characteristics from the leaf base towards the apex adjacent to the midrib. (a) leaf thickness, (b) the ratio of palisade to sponge tissue thickness (P/S ratio), (c) leaf mass per area (LMA), (d) chlorophyll concentrations per area, (e) guard cell length, (f) stomatal density, (g) minor vein length per unit leaf area (minor VLA). The X-axis represents the distance from the leaf base towards the apex within leaves (see Figure 1). Bars denote 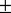
1 SE. Each mean value at each point was the average of six leaf discs.

**Figure 3:**
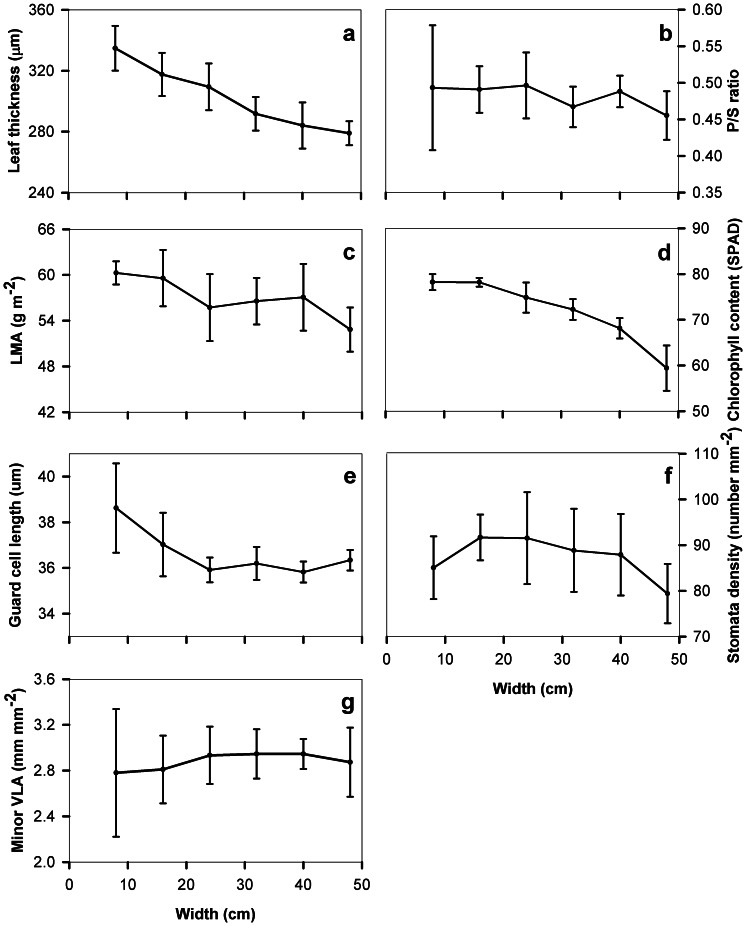
Leaf anatomical and structural characteristics from the leaf center to the outer regions adjacent to the secondary vein. (a) leaf thickness, (b) the ratio of palisade to sponge tissue thickness (P/S ratio), (c) leaf mass per area (LMA), (d) chlorophyll concentration per area, (e) guard cell length, (f) stomatal density and (g) minor vein length per unit leaf area (minor VLA). The X-axis represents the distance from the center to the outer regions within leaves (see Figure 1). Bars denote 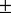
1 SE. Each mean value at each point was the average of six leaf discs.

**Figure 4:**
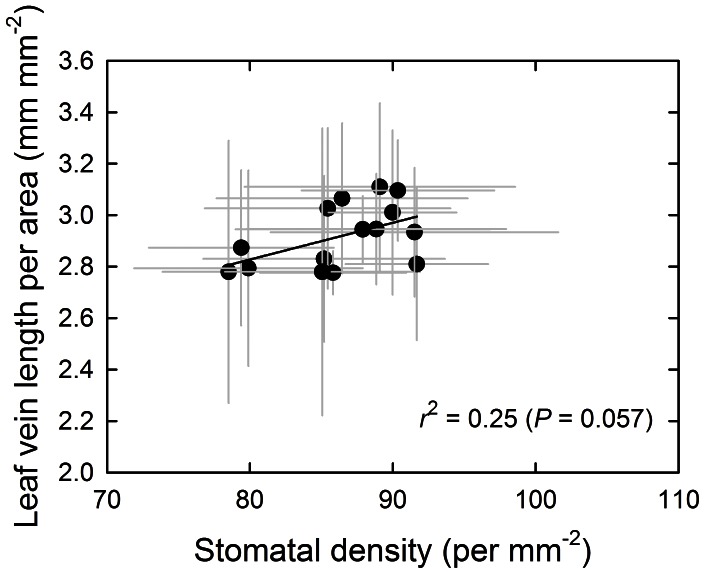
Correlations of stomatal density with leaf vein length per area (VLA) within leaves of *Alocasia macrorrhiza*. Data were fitted by linear regression. Error bars indicate ±1 SE.

We found strong positional variations in anatomical characteristics from the base towards the leaf apex and from the central to the outer regions (Figs 2 and 3). From the leaf base towards the tip, leaf thickness continuously decreased by 25% and from the center to the outer regions by 17% (Figs. 2a and 3a). Guard cell length tended to decrease and stomatal density to increase from the base towards the tip, by 9–10% (Figs. 2e and f). In contrast, leaf dry mass per area, chlorophyll concentration per leaf area, minor vein length per area and the ratio of palisade- to spongy- tissue thickness remained stable from the leaf base to leaf tip. From the midrib to the margin, chlorophyll concentration per leaf area decreased by 24%, leaf dry mass per area decreased by 12%, and stomatal density by 7%, and guard cell length 3% (Fig. 3). Minor vein length per area remained stable from the midrib to the lateral margin, varying between 2.8–3.1 mm mm^−2^ (Figs. 2 and 3). Leaf minor vein length per area showed a non-significant empirical trend to increase with stomatal density (Fig. 4).

### Within-leaf Variation in Physiology

**Figure 5:**
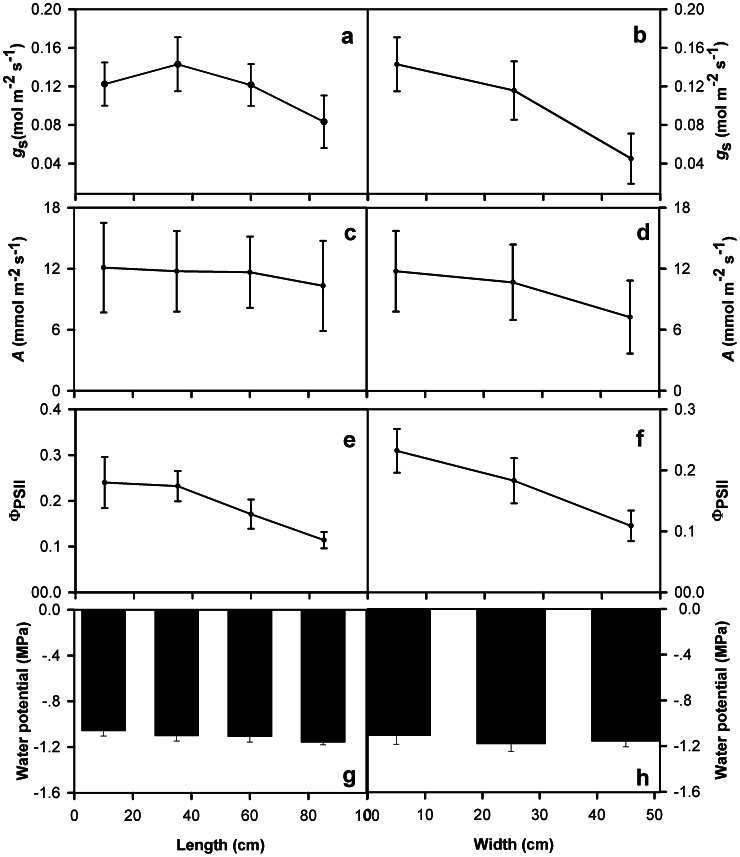
Leaf physiological characteristics at midday on sunny days from the basal area towards the leaf apical area adjacent to the midrib and from the center to the outer region and adjacent to the secondary vein. (a, b) stomatal conductance to water vapor (*g*_s_), (c, d) CO_2_ assimilation per leaf area (*A*), (e, f) the quantum yield of PSII (Φ_PSII_), (g, h) leaf water potential. The X-axis indicates the distance from the leaf base towards the tip (left panels) or from the middle vein towards leaf lateral margin (right panels) as illustrated in Figure 1. Bars denote 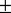
1 SE. Each mean value at each point was the average of six leaf discs.

From the leaf base to the tip, midday stomatal conductance, CO_2_ assimilation per leaf area and quantum yield of PSII decreased by 32%, 3% and 53% respectively (Figs. 5a, c and e) and declines were also observed from the midrib to the lateral leaf outer regions by 69%, 38% and 53% respectively (Figs. 5b, d and f). However, no positional variation in midday leaf water potential was found; values were approximately −1.2 MPa across the leaf (Figs. 5g and h).

### Within-leaf Variation in Temperature

**Figure 6:**
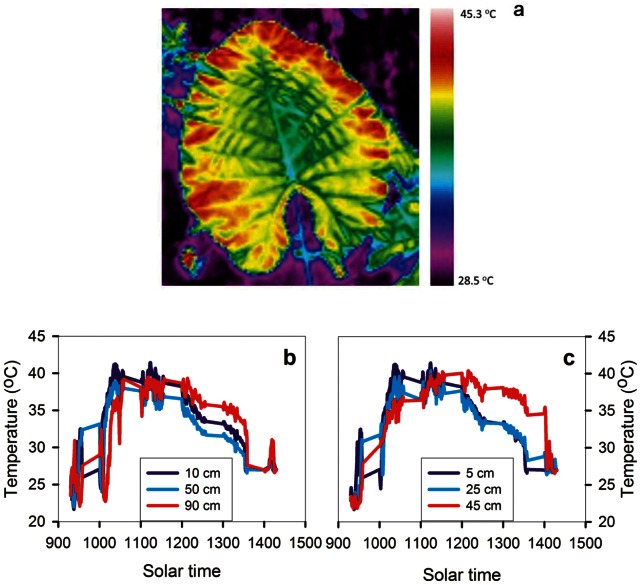
Thermal colour images and the diurnal time courses of leaf temperature in an *Alocasia macrorrhiza* leaf on a clear day during the rainy season. In panel a, the different colors represent the differences of temperature within a whole leaf. In panels b (along the midrib) and c (along the secondary vein), the numbers with different colors indicate the distance from the leaf base towards the tip (left panels) or from the middle vein towards leaf lateral margin (right panels) within leaves. Numbers are the distance at the two directions.

The sun-exposed leaves showed strong diurnal and positional influences on leaf temperature, and the variation for a typical leaf is shown in Figure 6. In the morning, the temperatures of the outer regions were lower than that of the center regions (Fig. 6), probably because of greater transpiration at the edge due to the lower boundary layer conductance, and potentially also due to a lower light interception in the outer regions owing to smaller leaf thickness (Fig. 3a) and higher leaf positional deviations from the direction of solar radiation in the outer region (S Li, *unpublished observation*). These subtle difference, however, were reserved at mid-day, in which a remarkable variation developed in midday leaf temperature between the center and the outer leaf regions in response to the higher temperatures and irradiances at mid-day (Figs. 6a–c). A relatively low leaf temperature was maintained around the midrib region in the afternoon. Leaf temperature in the outer regions reached 40°C during daytime, about 8.8°C higher than at the central region, and this spatial patterning was maintained for approximately two hours between 1200 h and 1400 h. This pattern was seen in both the thermal imaging and thermocouple data (Fig. 6). This spatial pattern in leaf temperature in the afternoon ran counter to that expected from the thinner boundary layer in the outer than in the center region and/or the lower light interception in the thinner outer regions, because those effects would be expected to reduce temperature at the outer leaf regions relative to the center. However, the observed pattern was consistent with the higher stomatal conductance observed in the central lamina than the tip and outer regions, indicating higher transpiration rates and greater cooling in the central regions.

**Figure 7:**
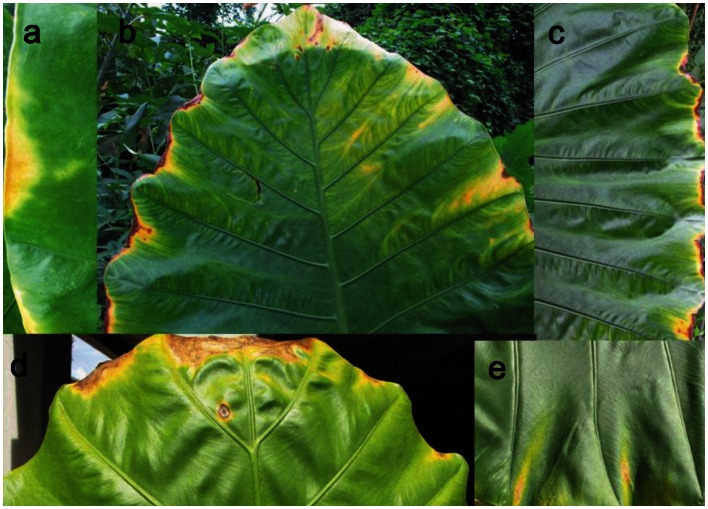
Illustrations of leaf dieback in the marginal areas of *Alocasia macrorrhiza*. Photos by S. Li and W.-L. Zhao.

Leaf dieback was very frequently observed for sun-exposed leaves at leaf tips and lateral margins especially between secondary veins where the lamina is most exposed (Fig. 7). No such leaf dieback was observed in shaded leaves.

## Discussion

As hypothesized, we found a strong heterogeneity in structure and physiology across giant leaves in *Alocasia macrorrhiza*.

In general, the patterning of traits across the leaf surface was consistent with reduced water supply and demand from midrib to outer regions and scaled back CO_2_ assimilation at mid-day. Thus, from the lamina center to outer regions, leaves become thinner, with lower leaf dry mass per area and chlorophyll concentration per area, and lower stomatal size and densities, and thus a reduced stomatal pore area for gas exchange. The lower stomatal conductance, CO_2_ assimilation and quantum yield of PSII at outer lamina regions in the afternoon were consistent with this pattern. The reduced stomatal conductance can explain the maintenance of homogeneous water potential across the leaf despite a lower intrinsic hydraulic supply at the outer relative to inner regions, or greater extrinsic stress at the outer regions.

An explanation for the lower stomatal conductance at the outer regions may be a lower water supply due to hydraulic limitation if water transport to the outer regions involves greater hydraulic resistance. Leaf vein length per area is a trait that contributes strongly to leaf water transport capacity, increasing the xylem pathways for water flow and reducing the distance water must travel outside the xylem [31], [32], [33]. Even though a similar minor vein length per area was found across the leaf surface, the further from the lower-order veins, the greater the distance water must travel in minor veins, and thus the greater the hydraulic resistance to that location [34], [35], [36].

The lower stomatal conductance at the outer leaf regions at mid-day leading to higher temperatures contrasted with the opposite pattern observed in the mornings, in which lower air temperatures and irradiances resulted in a lower evaporative load. The boundary layer of still air is thinner at the leaf outer regions under low wind speeds [37], and the outer regions would experience a greater transpiration load at a given stomatal conductance, as observed in the morning. However, at mid-day, the lower transpiration rate at the edges, indicated by the higher temperature, as well as the gas exchange measurements, suggested that, in fact, the reduction of stomatal conductance was greater than necessary to compensate for the thinner boundary layer. Thus, there was a true reduction of transpirational demand, likely compensating for the greater hydraulic resistance of water transport to the outer regions. The strong depression of stomatal conductance observed in the outer region would help conserve water and maintain water potential, but at the cost of transpirational cooling. Consistent with these principles, the lamina close to the primary vein were well-supplied hydraulically and maintained a relatively low temperature (Fig. 6). The reduced transpirational demand observed at the leaf outer regions was also associated with a reduction in mesophyll internal surface area and leaf thickness, as well as chlorophyll, and photosynthetic efficiency (and thus photosynthetic rate).

The gas exchange measurements across the leaf required cutting of the leaf lamina, though we avoided cutting major veins. One concern with this design is the possibility of effects of leaf cutting on stomatal aperture. However, the evidence of leaf temperature from the thermal imaging and thermocouple measurements was well-aligned with, and supported the pattern shown by the gas exchange measurements, both supporting higher temperature and lower gas exchange in the leaf outer regions. Previous experiments with leaf cutting treatments on many species have shown that if major veins are not severed, the lamina water potential and stomatal conductance does not change relative to surrounding areas over a time period of days [38], [39], [40]. However, cutting has been found to influence stomatal aperture on a shorter time scale, if the xylem was severed-e.g., if leaves were excised, or if the midrib were cut. Indeed severing the hydraulic supply typically causes a transient stomatal opening, as found when leaves are excised, either due to a release of xylem tension, leading to recovery of water potential in the guard cells (the “Ivanov effect”), though this would not be expected if the xylem is cut in air, or, alternatively, stomata may open due to a stronger tension in the xylem leading the epidermal pavement cells to dehydrate during ongoing transpiration, causing stomata to gape temporarily, until the guard cells can adjust osmotically [41], [42]. However, such mechanisms for transient stomatal opening would not be expected to arise here, given that the xylem supply to the lamina (main veins) were not severed. Thus, because two lines of evidence (thermal imaging, and gas exchange measurements on cut leaves) supported the same patterns of gas exchange heterogeneity across the leaf, and because the major veins were leaf intact, the experiments can be considered as providing strong support for lower stomatal conductance and transpiration in the outer leaf regions.

Notably, the question of how leaf physiology and structure vary across the leaf for species with leaves of different sizes has not been systematically considered to our knowledge. Larger leaves generally have a thicker boundary layer in their central regions than smaller leaves [12], [43], [44]. This thicker boundary layer is assumed to result in a lower transpiration rate and thus a higher heat load in larger leaves. In very larger leaves, the increased heat load coupled with increased hydraulic path length and decreased water supply to the outer regions of large leaves might be expected to lead to a greater spatial heterogeneity in structure and physiology across the leaf surface in larger leaves. While the ideal study of this question would apply the same methodology to species ranging in leaf size, relating the data from our study with those from previous studies on smaller-leafed species allows a first comparison. This comparison suggested that the variation in physiology may be stronger across the large leaf of *A. macrorrhiza* than for smaller leafed species, whereas the variation in internal and external structural traits appears to be within the range of that observed for smaller-leafed species. For stomatal conductance and CO_2_ assimilation per leaf area, values varied by up to 69% and 38% across the leaf for *A. macrorrhiza*. By comparison, a previous study of *Helianthus annuus* found a 20% decrease of stomatal conductance from base to tip [45], but in other studies of small-leafed species, no substantial differences were found across the lamina. By contrast, in grasses, often the tip had a higher stomatal conductance and CO_2_ assimilation per leaf area than the base, likely related to its greater light exposure [16], [46], [47], [48]. For quantum yield of PSII, values varied by up to 53% across the leaf for *A. macrorrhiza*. By contrast, for *Arabidopsis thaliana* there was little difference across the leaf surface for well-watered plants [49], though leaves on droughted plants showed strong declines from the leaf base to the tip. The stability of leaf water potential across the leaf observed for *A. macrorrhiza* in our study, was also found for tobacco [16], though in grasses in which the center of the leaf is most sun exposed, gradients of leaf water potential can mirror those of transpiration [50]. Further, in our study, temperature varied by 8.8°C across the leaf surface for *A. macrorrhiza*, whereas previous studies have found only a slightly lower transpiration from base to tip in the soybean leaflet, resulting in a 2°C lower temperature [38].

For structural features, values across the leaf surface were not strikingly heterogenous in *A. macrorrhiza*. For stomatal density, values varied across the leaf surface by 9%, while studies of smaller-leafed species showed variable trends in whether values increased or decreased or were stable among basal, central and apical leaf regions, with variation of up to 50% among regions [16], [48], [51], [52], [53]. Similarly, the decline of guard cell length from base to tip in *A. macrorrhiza* was moderate and opposite in direction relative to studies on smaller leaves, which have tended to show increasing values from leaf base to tip by up to 20% [48], [52]. The relative stability of vein density across the *A. macrorrhiza* from midrib to outer regions was especially noteworthy. In one previous study of the tobacco leaf, a slight increase from base to tip was reported [16]. However, for a range of grass species, vein length per area showed a slight to very substantial (up to 67%) decrease from base to tip [16], [48], [54], [55]. The decline in leaf thickness and leaf dry mass per area from base to tip in *A. macrorrhiza,* 25% and 12% respectively, were comparable with those reported for smaller-leafed species, which ranged from very slight [56] or up to 50% in a grass [55], [57]. Future comparative studies are needed of the variation of chlorophyll concentration and palisade: spongy ratios across the leaf in other species.

The dieback that is frequently observed at the edges of giant leaves may be analogous to that observed in terminal branches due to hydraulic limitations in tall trees [58], [59], [60]. The dieback at the edges of the giant leaf could thus result from reduced water supply to the outer regions, caused by an increase in hydraulic resistance as the xylem conduit diameters decrease and hydraulic path-length from the major veins increases [61], [62], [63]. Another hypothesis for dieback at leaf edges is the effect of toxic contents of guttation fluids driven by root pressure out of hydathodes in the leaf margins. Thus, previous studies suggested that damages in the leaf margins of banana leaves [64], [65] can occur due to the concentration of ions, including boron, in guttated xylem sap. However, this mechanism appears less likely to explain the damage observed in *A. macrorrhiza*. The leaf margin dieback was observed for sun-exposed leaves, but not for individuals in the shade, though plants in both high and low irradiances produce high root pressure (S Li, *unpublished data*).

Based on these findings, one might expect that the reduction of function and dieback at the outer edges of leaves, acting to maintain an even leaf water potential, might place a limit on the size that leaves can achieve for a given hydraulic supply system. According to this hypothesis, leaves could only develop to a size that will be functionally supplied with water. Indeed, smaller leaves tend to occur in drier and more open habits [43], [66]. Although larger leaves have benefits for absorbing diffuse light for a given mass investment in leaf surface [67], their thicker boundary layers compared to small leaves would then lead to stomatal closure and reduced convective cooling and gas exchange [43], [68]. Such leaves generally require larger major veins for support, containing greater xylem conduit numbers and sizes [12], [13], [69], but the major veins are spaced further apart in larger leaves, resulting in a lower major: minor vein ratio, which would leave the outer areas of the leaf with a lower hydraulic supply [13], [70], [71], [72].

In conclusion, our results indicate a remarkable heterogeneity of structure and function across giant leaves of *A. macrorrhiza*. Leaves appear to function typically with diminished function in the marginal areas, and giant leaves have especially steep within-leaf gradients in their structure and physiology.

Like previous studies of heterogeneity of structure and function across leaf surfaces, our study supported this hypothesis in suggesting that leaves are constructed with hydraulic limits at their edges. Future work is necessary to determine whether under higher transpiration loads, or drought, the edges of the leaves reach limits of thermal tolerance or dehydration. In that case the size of the leaf may be controlled by the design of the vascular system, i.e., by the densities and conductivities of its leaf veins. Such an expectation is supported by previous studies showing that among closely-related species, larger leaves tend to have greater cross-sectional conductivities in their major veins [69], [73], [74]. The strong heterogeneity across the leaf surface in giant leaves may present a major disadvantage to their evolution, a hypothesis which requires testing across a wide range of large-leafed species. Such a disadvantage could contribute to explaining the relative rarity of giant-leafed species globally, even in very resource-rich environments.
